# A study protocol for testing the feasibility of a randomised stepped wedge cluster design to investigate a Community Health Intervention through Musical Engagement (CHIME) for perinatal mental health in The Gambia

**DOI:** 10.1186/s40814-019-0515-5

**Published:** 2019-11-07

**Authors:** Katie Rose M. Sanfilippo, Bonnie McConnell, Victoria Cornelius, Buba Darboe, Hajara B. Huma, Malick Gaye, Paul Ramchandani, Hassoum Ceesay, Vivette Glover, Ian Cross, Lauren Stewart

**Affiliations:** 1 Goldsmiths, University of London, London, UK; 2 The Australian National University, Canberra, Australia; 3 Imperial College London, London, UK; 4 The Ministry of Health and Social Welfare, Banjul, The Gambia; 5 The National Centre for Arts and Culture, Banjul, The Gambia; 6 University of Cambridge, Cambridge, UK

**Keywords:** Perinatal mental health, Feasibility trial, The Gambia, Music, Singing group, Kanyeleng

## Abstract

**Background:**

Perinatal mental health problems affect up to one in five women worldwide. Mental health problems in the perinatal period are a particular challenge in low- and middle-income countries (LMICs) where they can be at least twice as frequent as in higher-income countries. It is thus of high priority to develop new low-cost, low-resource, non-stigmatising and culturally appropriate approaches to reduce symptoms of anxiety and depression perinatally, for the benefit of both mother and child. Music-centred approaches may be particularly useful in The Gambia since a range of musical practices that specifically engage pregnant women and new mothers already exist.

**Methods:**

This protocol is for a study to examine the feasibility of undertaking a stepped wedge trial to test how a Community Health Intervention through Musical Engagement (CHIME) could be beneficial in alleviating perinatal mental distress in The Gambia. In this study, we plan to recruit 120 pregnant women (*n* = 60 intervention, *n* = 60 control) at four antenatal clinics over two 6-week stepped sequences. Women in the intervention will participate in weekly group-singing sessions, led by local Kanyeleng singing groups, for 6 weeks. The control group will receive standard care. We will assess symptoms of anxiety and depression using the Edinburgh Postnatal Depression Scale (EPDS) and the Self-Reporting Questionnaire (SRQ-20). The feasibility of the design will be assessed through recruitment, retention and attrition rates of participants, clinics' adherence to the schedule and completeness of data by site. Qualitative interviews and video and audio recordings will be used to evaluate the acceptability of the intervention.

**Discussion:**

This feasibility trial will allow us to determine whether a larger trial with the same intervention and target group is feasible and acceptable in The Gambia.

**Trial registration:**

Retrospectively registered (24/01/2019) with Pan African Clinical Trials Registry (PACTR): PACTR201901917619299.

## Background

Perinatal mental health problems affect up to one in five women worldwide [1, 2]. Stress, anxiety and depression in pregnancy affect not only the mother but can also have long-term adverse effects on her child via biological mechanisms in utero [3]. Along with the impact on the mother and her developing infant, antenatal depression and anxiety are the most common predictors of postnatal depression [4, 5]. Postnatal depression can reduce her ability to provide sensitive and responsive caregiving that can potentially impair child development [6]. Mental health problems in the perinatal period are a particular challenge in low- and middle-income countries (LMICs) where they can be at least twice as frequent as in higher-income countries [1]. Our geographical context for this work will be The Gambia, in West Africa, where mental health services are minimal, services for perinatal mental health are non-existent and high levels of stigma associated with mental health issues, as well as specific local attitudes and beliefs, impede recognition and prevent help-seeking behaviour. It is thus of high priority to develop new low-cost, low-resource, non-stigmatising and culturally appropriate approaches to reduce symptoms of anxiety and depression perinatally, for the benefit of both the mother and child.

The current project will test the hypothesis that the creative arts—in particular group-singing—will show special promise in alleviating perinatal mental distress in The Gambia. In high-income countries, such as the UK and the USA, singing in groups has been shown to be a powerful modulator of mood and emotion, evoking positive effects on mental health, well-being and social affiliation [7] via mechanisms involving synchrony and entrainment [8], the saliency of relational communicative features in musical interaction [9] and significant effects on the endocrine system [10]. In addition, the mother’s voice is a key channel through which meaningful, sensitive and contingent interactions between the caregiver and infant can take place [11]. Recent studies have found that music and its use specifically during the perinatal period can reduce women’s stress levels and depressive symptoms and increase women’s attachment to their infant [12–15]. Music-centred approaches may be particularly fruitful in The Gambia as there are already a range of musical practices that specifically engage pregnant women and new mothers [16]. For instance, infant naming ceremonies occur 7 days after birth and are musical celebrations to recognise the new mother and her family [16]. Performances by Kanyeleng groups are closely associated with pregnancy and motherhood and are important in health communication [17]. These pre-existing cultural and creative practices provide an excellent context from which to explore, co-design and ultimately evaluate culturally situated, music-centred interventions that aim to reduce symptoms of anxiety and depression perinatally and facilitate mother-infant caregiving.

### Study aims

This is a feasibility study which aims to inform the design of a larger trial to investigate a Community Health Intervention through Musical Engagement (CHIME) to help reduce symptoms of anxiety and depression in pregnant women compared to standard care. This article describes the trial protocol (version 1.0, 11/11/18). The protocol was prepared in accordance with the Standard Protocol Items: Recommendations for Interventional Trials (SPIRIT) guidance. The trial SPIRIT checklist can be viewed in Additional file 1.

### Objectives

Our primary objective is to test (a) the feasibility of delivering a group-singing intervention to a sample of pregnant women in The Gambia using a stepped wedge design and (b) the feasibility of using standardised tools to measure the impact of this intervention on anxiety and depression symptoms, before and after the intervention.

This objective can be broken down into five specific feasibility objectives:

1. To obtain demographic information on the eligible population
2. To determine if our measurement tools, the Edinburgh Postnatal Depression Scale (EPDS) and the Self-Reporting Questionnaire (SRQ-20), are useable
3. To determine if the intervention is deliverable
4. To determine if the stepped wedge trial design is deliverable and obtain information that will inform the definitive study. Specifically to:
  - Assess recruitment and acceptability of randomising clinics
  - Assess the recruitment rate of women to control and intervention groups
  - Assess participants’ adherence to the intervention group and follow-up in both arms
  - Test the feasibility of data collection
5. To determine if this type of intervention is culturally appropriate and well received by the community and health workers.

## Methods/design

We will be testing the feasibility of a stepped wedge cluster design, which differs from a parallel arm cluster design in that all clinics involved in the study receive the intervention [18]. Advantages over a parallel arm cluster trial include the requirement of a smaller sample size due to the availability of a within group comparison and prevention of potential disappointment for health clinics who are not randomised into the intervention.

### Study setting

This multi-site study will recruit from four antenatal clinics in western Gambia.

### Study population

All participants will be Mandinka or Wolof Gambian women who are pregnant.

Inclusion criteria

- Pregnant (14–24 weeks gestation)
- Speak Wolof or Mandinka fluently

Exclusion criteria

- At least one previous late term miscarriage
- Current psychosis or history of psychosis

Withdrawal criteria

If the participant develops any serious medical condition or the participant’s mental health significantly declines (as assessed by the care team), and the care team deem it necessary, then she may be withdrawn from the study.

### Intervention

The intervention will be delivered on the community level, meaning that it will include women with a range of anxiety and depression symptoms. Our primary aim is to reduce symptoms in those experiencing them (whether these are at a high level or a medium or low level). We anticipate this may also help to reduce their symptoms into the postnatal period. By including those with low and high levels of symptoms, rather than screening and including only those with high levels of symptoms, we will aim to avoid stigma and increase acceptability.

The intervention has been developed following focus groups with various stakeholders including health professionals (midwives and community birth companions), pregnant women and musicians (griots and Kanyeleng groups).

Four groups of 20 women between 14 and 24 weeks gestation will attend six 60-min group-singing sessions at their local antenatal clinic. This will take place in the morning as this is the time deemed to best suit the majority of women and clinics. Local Kanyeleng groups who specialise in musical practices to support women’s health will lead the sessions. The content of the six sessions will be co-designed with the Kanyeleng groups via two extended workshops with the research team. All sessions will begin with a welcome song and end with a closing song. Some of the songs used during the main body of the session will cover topics including the (a) importance of the singing group in supporting each other, (b) importance of other positive relationships in their lives, (c) resilience to challenges and empowerment and (d) importance of being open, removing stigma to discuss challenges. One lullaby will be introduced at each session. Kanyeleng leaders will also be encouraged to ensure that all the women feel comfortable and are participating when they can.

The nature of the intervention will necessarily vary somewhat across the four settings, especially as Wolof speaking groups and Mandinka groups have different and distinct cultural beliefs, practices and language. By using the Kanyeleng groups local to each of the four clinics involved, the sessions will be contextually appropriate, while the workshop with all four Kanyeleng groups before the intervention begins will ensure that the overarching goals, content and approach to session delivery are broadly standardised.

Over the course of the 6-week intervention period, a research assistant will observe and video and audio record two singing sessions (the first and the fourth sessions) from each of the four clinics to ascertain, using a checklist, the extent to which the sessions conform to our articulated goals. A community health nurse at each clinic will be engaged to take attendance data and report any issues of concern to the research team.

The control group will consist of four groups of 20 women between 14 and 24 weeks gestation from the same four clinics. These women will receive only standard care without any additional intervention.

### Randomisation and blinding

As we will be testing the feasibility of delivering a stepped wedge cluster design, the four different antenatal clinics will be randomised with two sites starting first (creating the first sequence) and two starting 6 weeks later (the second sequence). Randomisation will be performed by the study statistician who will generate a randomisation list using software and apply it to the pre-concealed list of clinics. The researchers and participants will not be blinded to whether they are in the intervention or the control cohort.

### Outcome measurements

Two local research assistants (RAs) will collect all measures orally as there is a high rate of illiteracy among the target population. All scales have been translated into Mandinka and Wolof. The translation method used was based on suggestions by the World Health Organisation, Hanlon et al. [19] and Cox, Holden and Henshaw [20]. First, the scales were translated into Mandinka and Wolof. An expert panel discussion then refined the translation before back translating it into English. The expert panel came together once more to resolve any remaining issues before finalising the translation.

Two questionnaires will measure antenatal anxiety and depression symptoms. The Edinburgh Postnatal Depression Scale (EPDS) [21] is a ten-item scale that was developed to screen for postnatal depression. It has subsequently been validated to be used during pregnancy [22]. This measurement tool has been used and validated in other African contexts; however, there is no avaliable validated version of the EPDS in Mandinka or Wolof even though it has been used in The Gambia before [23]. The Self-report Questionnaire (SRQ-20) [24] is a 20-item scale developed by the World Health Organization to measure anxiety and depression symptoms in a variety of cultural contexts. It has been used in many different African contexts such as Ethiopia [19], South Africa [25] and Rwanda [26] and as a way to measure perinatal mental health [23]. However, the SRQ-20 has never been used in the Gambian context nor has it been translated into Wolof or Mandinka.

### Demographic outcomes

We will collect demographic information about all participants. The data we will collect is as follows: date of birth, gestational age, time taken to get to the health centre, parity, gravida, place of birth, current place of residence, ethnic group, history of serious illness, occupation, husband’s occupation, marital status, educational background and amount of regular musical engagement.

### Feasibility outcomes

The feasibility outcomes are as follows:

- Recruitment rate
- Retention and attrition rates of participants
- Clinics' adherence to stepped wedge schedule
- Completeness of data by site and over time
- Video and audio recording of sessions to determine fidelity of the intervention at each site, i.e. whether key content emphasised in training workshops was being delivered at each site.
- Qualitative interviews with participants after the intervention to capture enjoyment and perceived benefit.

### Recruitment

Four antenatal clinics will be chosen to take part in the study based on three criteria: (1) availability of a local Kanyeleng group to deliver the intervention, (2) the language group predominantly spoken in the area (with at least one clinic being in a predominantly Wolof speaking area) and (3) the type of community the clinic serves (with at least one within an urban area).

The health professionals working at the health centre will first approach participants for the study. If they meet the criteria, they will be given information about the study and asked if they would like to be referred to the RAs. They will then be put in contact with one of the RAs who will meet them privately face to face. The information sheet will be read out in their native language verbatim to ensure participants’ ability to give informed consent. Consent will be taken orally by the RAs and recorded by signature or thumbprint. If participants do not choose to take part we will record their reason, if it is given, to help understand why women might not want or be able to participate.

### Incentive and participant retention

Participants in both groups will be offered a total of 600 Dalasi (about 12 USD) for their time, 200 Dalasi for each of the three data collection time points (baseline, post, follow-up). All participants will be reminded of the data collection and the group-singing sessions by phone call. Calls will be made by the RAs 3 days and 1 day before as well as on the day of these appointments. Where possible, a record will be kept of the reasons women give for failing to join the intervention or data collection session.

### Sample size

As this is a feasibility study, it is not designed to assess the efficacy of the intervention, although pilot data on this will be collected. We will evaluate the feasibility of study design, data collection and whether the intervention is deliverable and acceptable to the participants. In the study, we will gather information to be used in the design of the future definitive study including an estimate of the standard deviation of potential primary outcomes to inform the sample size. We aim to collect data from a total of 120 pregnant women, 60 in the control condition and 60 in the intervention condition [27]. This number will be sufficient to provide estimates of binary feasibility outcomes with precision of at least ± 9 percentage points for the 95% confidence interval.

### Trial schedule

**Fig. 1:**
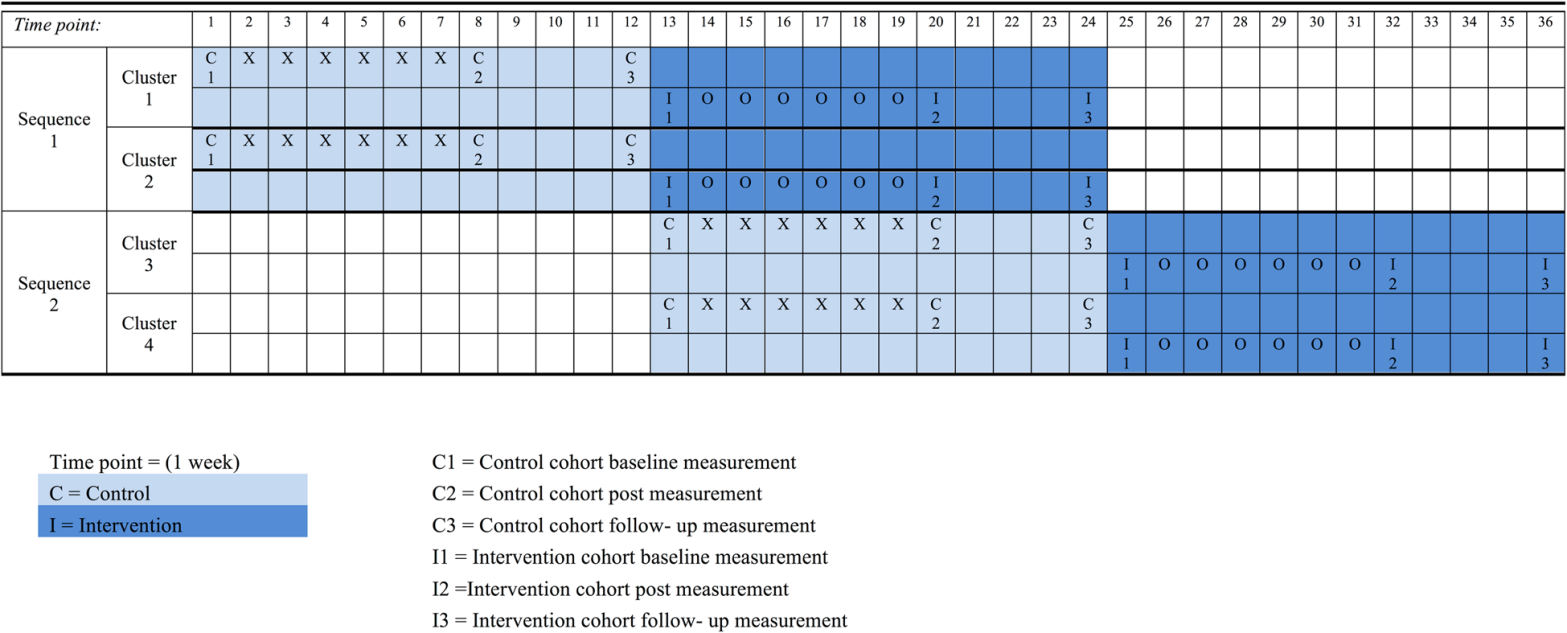
Schematic of the study

This trial design involves a sequential crossover of clusters whereby each cluster (antenatal clinic) receives the control condition followed by the intervention condition. The four chosen clinics will be randomised to two sequences of a 12-week phase. A separate cohort of participants will be recruited to the control group and the intervention (singing) group. Each cohort will be recruited around 4–6 months into their pregnancy. The 12-week phase for both the control and intervention cohort will include data collection at week 1 (“baseline”) and week 7 (“post”) after either group-singing (intervention) or standard care (control) as well as at week 11 (“follow-up”), 4 weeks after the intervention finished. Contamination will be avoided by having data for the control group collected before the intervention groups start at each clinic. See Fig. 1 for a schematic for the study.

### Assessment and management of risk

There are no high risks within our study compared to standard care. We have identified three areas of ethical concern and have outlined how each of these issues will be managed.

1. Mothers may experience an adverse effect such as a miscarriage, difficult birth, still birth, a sudden drastic change in physical or mental health, infant health problems or even a serious adverse effect such as death during the intervention. It is possible that a participant experiencing such an adverse event may attribute a causal link between the adverse event and their involvement in the study. We will mitigate this association being made, firstly, by clearly explaining the nature of the intervention and any possible risks to the women when they are recruited into the study. If, despite this, an association of this nature was still made, we would enlist the help of the Ministry of Health & Social Welfare (our partner on the project) to disseminate information to the women and the community concerning the incidence of such events occurring in the general population in an attempt to reassure those concerned that such adverse events should not be attributed to involvement in the study.
2. It is possible that some of the themes involved in questionnaires could lead to the women revealing episodes of self-harm. If this is the case, the woman will receive in the moment front-line counselling to talk through these issues with the RAs who are trained psychiatric nurses. Then, if needed, she will be referred on to the community mental health team (CMHT) for further management. If the CMHT deems it appropriate, they may then refer her on to the psychiatric team.
3. It is possible that some of the themes involved in questionnaires may also lead the women to reveal domestic abuse. If this is the case, the woman will receive in the moment front-line counselling to talk through these issues with the RAs. For emergencies and cases that require immediate intervention, the RAs will connect with the Gender-Based Violence focal person. For other cases, the RAs will refer the woman to the One Stop Center at Serekunda General Hospital or Edward Francis Small Teaching Hospital.
4. Women throughout the study will be monitored by the RAs, both trained psychiatric nurses. If the RAs feel that at any point a woman’s score indicates a high level of symptoms and/or the women reveal that they are particularly struggling, the RAs will refer the woman on to the Community Mental Health Team (CMHT) for further management. If the CMHT deems it appropriate, they may then refer her on to the psychiatric team.

### Data management

All consent forms will be stored in a master file, which will be kept in a locked drawer where only members of the research team have access. All case report forms will not be linked to names, just a participant number, and kept in a separate locked cabinet where only the research team has access. All data, including video and audio recordings, will be held on an encrypted hard drive only members of the research team can access. Data will be stored for 5 years after the study and will then be deleted or destroyed.

### Analysis

All data will be entered into a database by an RA and verified by the second RA using double data entry to ensure data quality. As this is a feasibility study, we will examine missing data as an outcome.

Descriptive statistics will be summarised to understand the demographic variables relating to the recruited population. Descriptive statistics and plots will be used to assess the distribution of the measurement tools, repeated at baseline and follow-up and by each arm. We will also examine the distributions of scores in the different language groups to see to what extent item scores and overall distributions differ or are similar. Correlations between our two measurement tools will be calculated.

To determine if the intervention is deliverable, we will record the number of sessions that the Kanyeleng groups delivered, aiming to deliver two thirds of the sessions, and the duration of each session, aiming to last between 45 and 75 min. We will also perform a qualitative evaluation, using the video and audio recordings, to determine intervention fidelity at the four sites. Both RAs will watch the video and audio recordings of the first and fourth group-singing sessions at each clinic and complete a checklist to determine if all the necessary elements—as outlined in the training workshops—were included in the intervention. Reliability of the fidelity measure will be ascertained by measuring inter-rater consistency.

We will also calculate the proportion of clinics approached that consented, aiming to reach over 50% recruitment rate, and record any scheduling problems in keeping with the stepped wedge timeline. Recruitment, adherence and completeness of data will be calculated for both groups. We aim to achieve a 60% recruitment rate and no more than 30% attrition in both arms. To determine if the intervention was culturally appropriate and well received by the community and health workers, we will collect qualitative data from post-intervention interviews and perform a thematic analysis.

## Discussion

The absence of mental health services in The Gambia, coupled with the stigma associated with mental illness in general, results in high levels of unmet need for pregnant women dealing with mental distress in The Gambia. The development of a low-cost, low-resource intervention, which is rooted in local health and cultural practices, is of high priority, and the feasibility study we intend to carry out will inform a full-scale trial to investigate efficacy of such an approach.

By employing local research assistants and creating a partnership with governmental agencies, such as The Ministry of Health & Social Welfare and The National Centre for Arts and Culture, this study brings the understanding of existing health services and access to a network of primary healthcare workers throughout the country as well as the diversity of local musical practices and the meanings attached to them. This valuable knowledge will help us cope with the practical and operational issues that may arise. We hope to disseminate our findings within various scientific publications, during field days in various areas in The Gambia, and during a meeting in London which will bring together the researchers as well community members, academic colleagues and health professionals interested in hearing about this work.

### Trial status

This article describes the protocol for a Community Health Intervention through Musical Engagement (CHIME) for perinatal mental health in The Gambia (version 1.0, 11/11/18). The sponsor for this trial is Goldsmiths, University of London. The project is funded by the MRC and the AHRC. Ethical approval was obtained from the Goldsmiths University Ethics Committee, the Research and Publication Committee (RePubliC) from the University of The Gambia and the Australian National University ethics committee.

## Supplementary information

**Additional file 1.** SPIRIT Checklist.

## Data Availability

All of the data will be held by the principal investigator, and the research team has exclusive use of the data until the publication of the results.

## Abbreviations

AHRC: Arts and Humanities Research Council
CMHT: Community mental health team
EPDS: Edinburgh Postnatal Depression Scale
LMICs: Low- and middle-income countries
MRC: Medical Research Council
PACTR: Pan African Clinical Trial Registry
RAs: Research assistants
SRQ-20: Self-Reporting Questionnaire
